# Landscape of adenosine pathway and immune checkpoint dual blockade in NSCLC: progress in basic research and clinical application

**DOI:** 10.3389/fimmu.2024.1320244

**Published:** 2024-01-29

**Authors:** Rulan Wang, Zhenkun Liu, Ting Wang, Jiabi Zhang, Jiewei Liu, Qinghua Zhou

**Affiliations:** ^1^Lung Cancer Center, West China Hospital, Sichuan University, Chengdu, Sichuan, China; ^2^Department of Medical Oncology, Cancer Center, West China Hospital, Sichuan University, Chengdu, Sichuan, China; ^3^Department of Nutrition and Integrative Physiology, College of Health, University of Utah, Salt Lake City, UT, United States

**Keywords:** adenosine, CD73, non-small cell lung cancer, immunosuppressive microenvironment, immunotherapy, drug resistance

## Abstract

Lung cancer poses a global threat to human health, while common cancer treatments (chemotherapy and targeted therapies) have limited efficacy. Immunotherapy offers hope of sustained remission for many patients with lung cancer, but a significant proportion of patients fail to respond to treatment owing to immune resistance. There is extensive evidence to suggest the immunosuppressive microenvironment as the cause of this treatment failure. Numerous studies have suggested that the adenosine (ADO) pathway plays an important role in the formation of an immunosuppressive microenvironment and may be a key factor in the development of immune resistance in EGFR-mutant cell lung cancer. Inhibition of this pathway may therefore be a potential target to achieve effective reversal of ADO pathway-mediated immune resistance. Recently, an increasing number of clinical trials have begun to address the broad prospects of using the ADO pathway as an immunotherapeutic strategy. However, few researchers have summarized the theoretical basis and clinical rationale of the ADO pathway and immune checkpoint dual blockade in a systematic and detailed manner, particularly in lung cancer. As such, a timely review of the potential value of the ADO pathway in combination with immunotherapy strategies for lung cancer is warranted. This comprehensive review first describes the role of ADO in the formation of a lung tumor-induced immunosuppressive microenvironment, discusses the key mechanisms of ADO inhibitors in reversing lung immunosuppression, and highlights recent evidence from preclinical and clinical studies of ADO inhibitors combined with immune checkpoint blockers to improve the lung cancer immunosuppressive microenvironment.

## 1 Background

Lung cancer is a malignant tumor that seriously threatens human life and health worldwide, with a high incidence and mortality rate, making it one of the most common malignant tumors in recent years (1). Chemotherapy and targeted therapies have limited efficacy in lung cancer; even after effective chemotherapy, the 5-year survival rate of patients with advanced stages is only approximately 10% (2, 3). Moreover, the emergence of resistance to targeted therapy inevitably occurs within a short period (4). In recent years, immunotherapy has led to breakthroughs in the field of lung cancer (5). The combination of platinum-based chemotherapy and immunotherapy has resulted in an improved 5-year overall survival rate in patients with advanced non-small cell lung cancer (NSCLC), reaching 19.3% for non-squamous NSCLC (2) and 18.4% for squamous NSCLC (3). However, bottlenecks have inevitably been encountered. Due to the development of immune resistance, a significant proportion of patients show almost no benefit from immunotherapy (6). Further, there is some data to suggest that the objective efficacy rate for patients treated with PD-1/PD-L1 inhibitors as a monotherapy is only approximately 12.5%, with poor efficacy in the remaining 87.5% of the population (7). As such, expanding the beneficiary population of immunotherapy and enhancing its therapeutic effect have become important topics of research in the field of immunotherapy.

Studies have shown that the presence of a tumor-induced immunosuppressive microenvironment is a critical bottleneck limiting the development of immunotherapy (8, 9); this environment is related to the number and status of tumor-infiltrating lymphocytes (TILs) in the tumor microenvironment (TME) (10). As such, the effective reversal of the immunosuppressive microenvironment and enhanced efficacy of immunotherapy remain pressing issues (11). The adenosine (ADO) pathway is known to be critical for the formation of an immunosuppressive microenvironment. As such, inhibiting the activity of this pathway may be a potential mechanism to effectively reverse ADO pathway-mediated immune resistance (12). In addition, ADO inhibitors in combination with immune checkpoint blockers may be effective as a potential new oncological treatment option to expand the beneficial population for immunotherapy. Several recent studies have addressed the broad prospects of the ADO pathway as an immunotherapeutic strategy (13). However, few researchers have systematically summarized the theoretical basis and clinical rationale for ADO pathway and immune checkpoint dual blockade in lung cancer.

## 2 Overview of the rationale for the ADO pathway in reversing immune resistance

### 2.1 ADO metabolism

**Figure 1:**
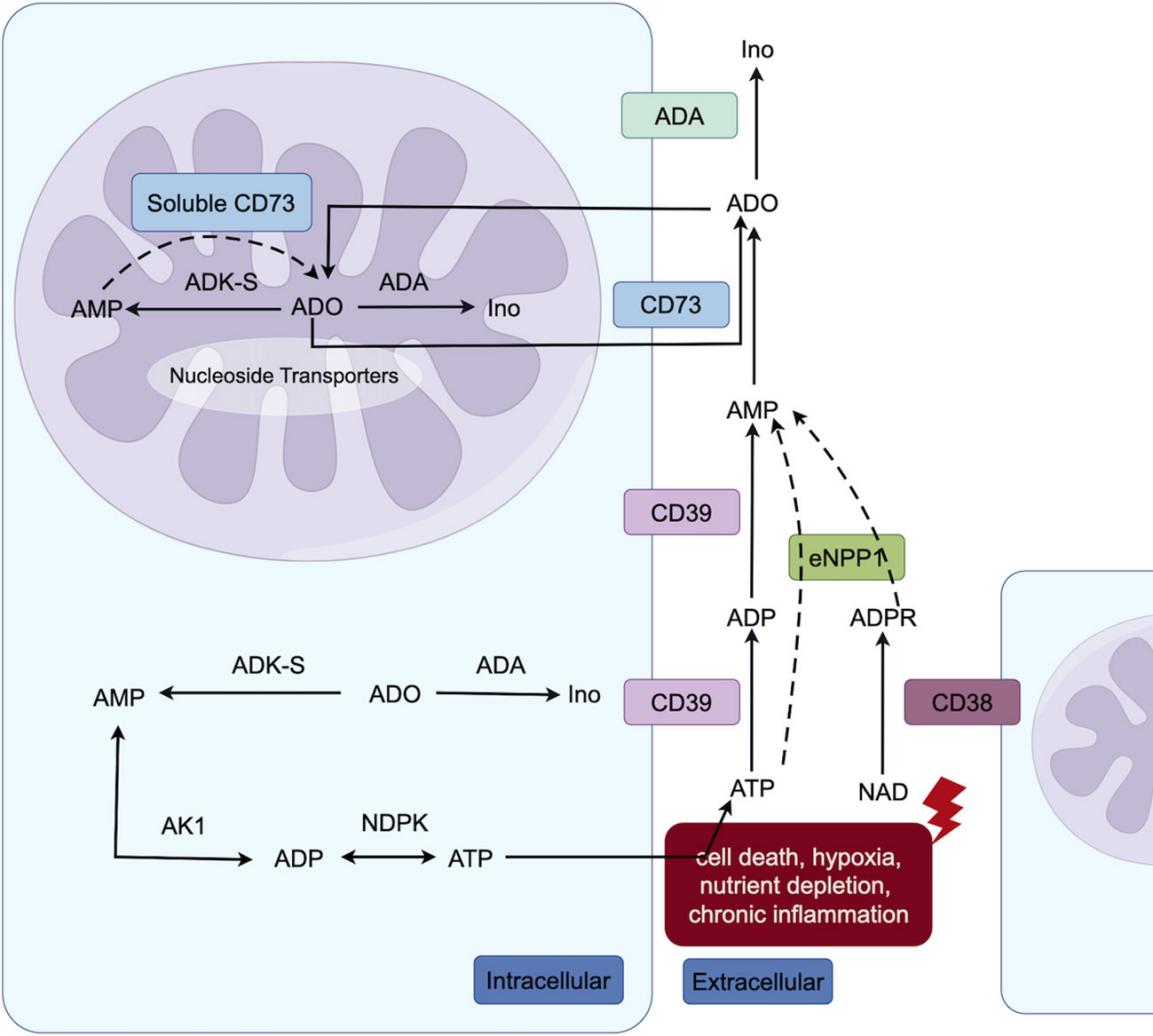
Intracellular (both cytoplasmic and nuclear) and extracellular adenosine metabolic pathways. Extracellular ATP undergoes conversion to ADO via the classical catabolic pathway, catalyzed by CD39 and CD73 enzymes. Additionally, nicotinamide-adenine dinucleotide is degraded by CD38 to generate adenosine diphosphate ribose, which can be further metabolized into AMP through the action of Extracellular nucleotide pyrophosphatase/phosphodiesterase-1, ultimately leading to ADO formation. Within the nucleus, AMP can also be converted into ADO by soluble CD73. Furthermore, both in nuclear and cytoplasmic, adenosine kinase can facilitate the synthesis of AMP from ADO, thereby participating in ATP metabolism (The figure is made by Figdraw).

Tumor cells, as well as other cells in the TME, actively secrete ATP in response to cell death, hypoxia, nutrient depletion, and chronic inflammation. Hypoxia and transforming growth factor-β promote solid tumors and immunosuppressive cells in the TME to express high levels of exonucleosidases (14). ATP is involved in numerous metabolic processes through various intracellular and extracellular pathways, ultimately leading to its conversion into ADO (**Figure 1**). In brief, the exonucleosidase CD39 hydrolyzes ATP to generate ADP and AMP, which are further converted to ADO by the exonucleosidase CD73 (13). The adenosine diphosphate ribose/cyclic adenosine diphosphate ribose generated by nicotinamide-adenine dinucleotide (catalyzed by exonuclease CD38) undergoes further metabolism by extracellular nucleotide pyrophosphatase/phosphodiesterase-1 to AMP, which serves as a substrate for ADO generated by exonuclease CD73. Consequently, CD38 plays a pivotal role in establishing an immunosuppressive tumor microenvironment in solid tumors. Notably, enzymes such as adenosine deaminase and adenosine kinase regulate the final metabolic conversion of ADO and the activation level of ADO receptors; however, their role in tumor development is unclear, making this a therapeutic target worth exploring in the ADO pathway (13).

### 2.2 Potential impact of ADO pathway inhibitors on the immune response in lung cancer

**Figure 2:**
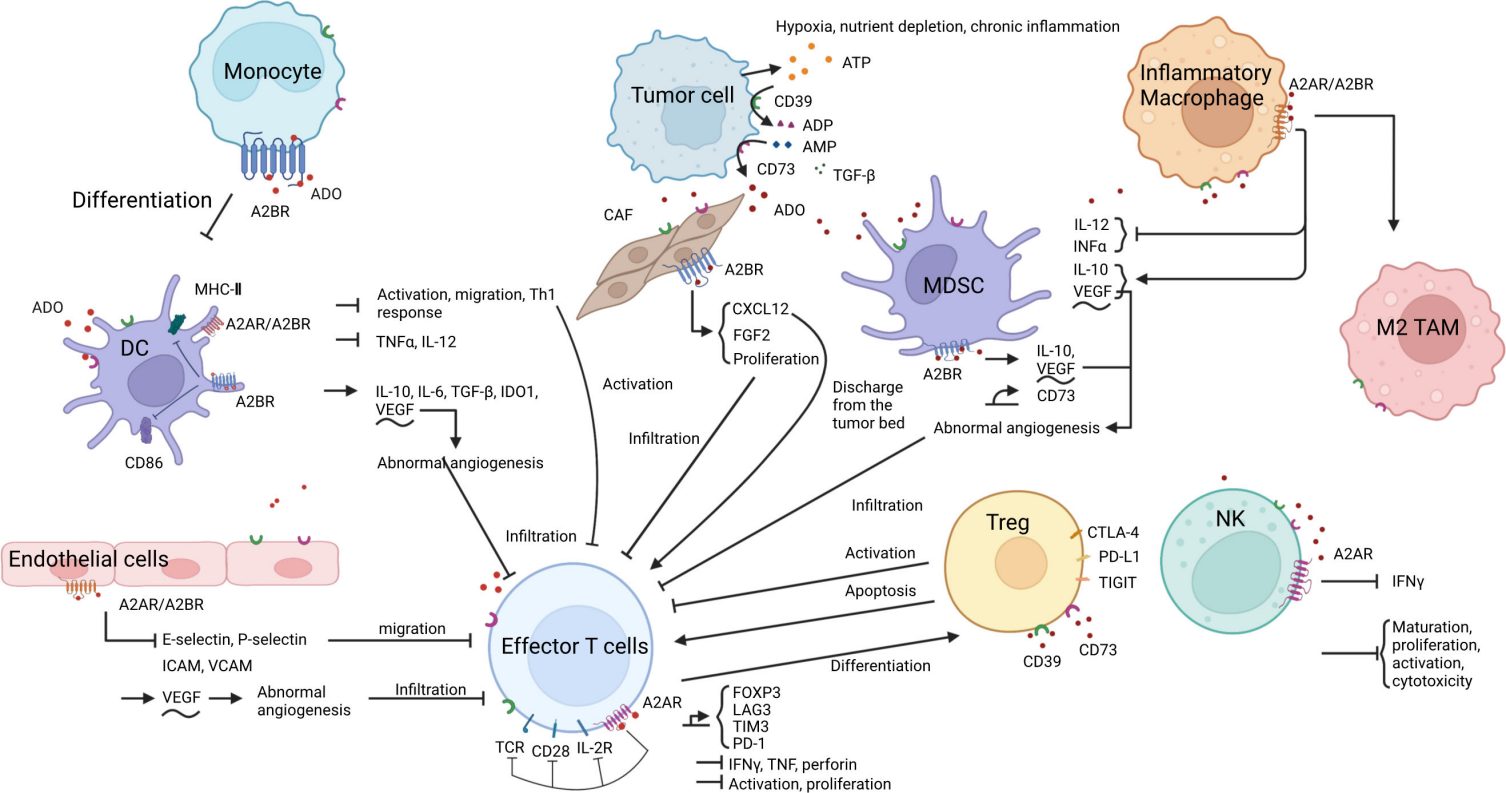
ADO and the formation of an immunosuppressive microenvironment. 1. ADO metabolism: Under specific conditions, ATP in the TME is converted into ADO by CD39 and CD73. 2. ADO-induced immunosuppression: ADO binds to A2AR or A2BR on the surface of various immune cells, endothelial cells, and fibroblasts, thereby eliciting diverse biological effects. These effects encompass inhibition of antigen presentation, impediment of immune effector cell activation, infiltration and function as well as NK cell activity, promotion of immunosuppressive cell proliferation and functional expression, induction of aberrant angiogenesis, ultimately culminating in the establishment of an inhibitory immune microenvironment.

Currently, the mechanisms of immunotherapy resistance include a lack of neoantigens or abnormal antigen presentation, low tumor load, low PD-L1 expression, T-cell infiltration disorder or T-cell exhaustion, presence of immunosuppressive cells or factors, and abnormal signaling pathways (15). This study therefore focused on elucidating the underlying mechanisms of resistance to immune checkpoint inhibitors associated with the ADO signaling pathway (**Figure 2**) as well as exploring the potential of ADO pathway inhibitors in overcoming immune resistance in lung cancer.

#### 2.2.1 Improvement of the immunosuppressive microenvironment

The ADO produced in the TME binds to A2 receptors (A2R) on monocytes (16), dendritic cells (DCs) (17), myeloid-derived suppressor cells (MDSCs) (18), regulatory T cells (Tregs) (19, 20), and macrophages (21, 22), thereby modulating their differentiation and function (16, 18–20, 23–26), ultimately leading to the induction of an immunosuppressive microenvironment conducive to lung cancer development (27, 28). Various inhibitors targeting the ADO pathway can improve the immunosuppressive microenvironment by inhibiting the process by which ADO acts, or by directly inhibiting ADO production (14). In the RAS mutant NSCLC mouse model, the novel CD73 antibody Ab001/Ab002 and the humanized antibody Hu001/Hu002 were found to effectively regulate the TME, reduce the infiltration level of M2 tumor-associated macrophages and MDSCs, induce the accumulation of mature DCs, promote effector T cells (Teff) proliferation and interferon γ (IFN-γ) secretion, enhance T-cell-mediated cytotoxicity, and ultimately inhibit tumor growth in mice (29). The proportion of Tregs in a co-culture system of lung adenocarcinoma cells and peripheral blood mononuclear cells was reduced following CD73 knockdown (30). The addition of AMP to the T cell *in vitro* culture system inhibited T cell proliferation and division, and this inhibition was alleviated by the addition of the anti-CD73 antibody oleclumab (30).

#### 2.2.2 Improvement of antigen presentation

The ADO receptor signaling pathway inhibits the activation of antigen-presenting cells (APCs) to hinder antigen presentation, thereby limiting the opportunity for T cell activation by antigens (16, 25, 31–33). ADO further impedes the migration of DCs (16), thereby preventing the encounter between antigen-carrying DCs and naïve T cells, and consequently regulating the immune response (34). Previous studies have demonstrated that myeloid ADO receptor A2 (A2AR) or ADO receptor B2 (A2BR) deficient mice exhibit elevated expression levels of costimulatory molecules CD86 and major histocompatibility complex II (MHC II, markers of the activation and maturation of antigen-presenting cells) on APCs, as well as increased CD8+ T cell activation and proliferation, higher levels of IFN-γ secretion on APCs, and slower tumor growth (25, 35). Additionally, A2R antagonists can reverse the impaired CD86 and MHC II expression in APCs (25, 32).

#### 2.2.3 Promotion of T-cell infiltration and function

ADO induces T cell infiltration disorder mainly by impairing the antigen presentation process (as described in the previous subsection) (10, 36) and inhibiting the secretion of various adhesion molecules such as e-selectin, p-selectin, and intercellular adhesion molecules (ICAMs) of endothelial cells to block T-cell migration (37) (38). Additionally, ADO can also promote abnormal angiogenesis by inducing vascular endothelial growth factor secretion, resulting in abnormal tumor vascular structure and function that hinder immune cell infiltration (14) (39). Furthermore, ADO in the TME binds to TILs (40, 41) and APCs (35), blocking effector T-cell activation, proliferation, and secretion of various cytokines such as IFNγ, tumor necrosis factor (TNF), and perforin (13, 38). ADO inhibitors direct normal T cell activation by improving antigen presentation and promoting the formation of a normal circulatory system, allowing activated T cells to enter the tumor bed with the assistance of the normal circulatory system (41). There is also evidence to indicate that Tregs in the TME inhibit the transendothelial migratory capacity of Teff by inducing high expression of CD39, promoting ADO production, and reducing monocyte-induced expression of the adhesion molecule ICAM-1 on endothelial cells (42). CD39 or ADO inhibitors effectively restore the migratory capacity of Teffs (42). ADO has also been shown to inhibit the chemotactic properties of CD3+ and CD8+ T cells by decreasing KCa3.1 channels. A2AR blockers or KCa3.1 channel activators can block this phenomenon and promote the migration and infiltration of T cells (43).

#### 2.2.4 Promotion of the secretion of IFN-γ

ADO has further been shown to block IFN-γ-induced STAT1 phosphorylation, inhibit the inflammatory response induced by macrophage activation (44), and eliminate the increased production of IL-12, IFN-γ, and TNF-α mediated by IL-18 (45). Treatment of activated CD4+ T cells with ADO resulted in a significant decrease in A2AR-mediated IFN-γ release (46). In contrast, the production of IFN-γ, TNF-α, and granzyme B was found to be increased in CD73-deficient cells, indicating an augmented cytotoxic potential (47). CD4+ T lymphocytes co-incubated with CD73 monoclonal antibody have increased IFN-γ production (48). Mutations in key genes in the IFN-γ signaling pathway result in loss of PD-L1-responsive expression, making such patients less likely to respond to PD-1 blockade therapy (49). It has been shown that anti-CD73 monoclonal antibodies can enhance the antitumor effects of PD-1 antibodies by promoting CD8+ T-cell infiltration and IFN-γ secretion (50). Caffeine is an A2AR inhibitor (50), and prior research has shown that the combination of caffeine and anti-PD-1 monoclonal antibodies can significantly increase the levels of TNF-α and IFN-γ in tumors, thus exerting stronger antitumor activity (51).

## 3 Preclinical and clinical evidence for reversal of immune resistance in lung cancer by the ADO pathway

Cases of NSCLC with epidermal growth factor receptor (EGFR) mutations are known to respond poorly to immune checkpoint inhibitor therapy (52). Although it has been shown that PD-L1, tumor mutation burden (TMB), and CD8+ TILs are all significantly higher in patients with resistance to EGFR-tyrosine kinase inhibitor (TKI) therapy (53, 54), this elevation does not seem to translate into a benefit in immune checkpoint blockade (ICB) treatment (55–58). The poor outcomes of these patients appear to be linked to Treg cell-mediated immunosuppression (59, 60). Le et al. (12) previously analyzed NSCLC samples from multiple databases at multiple levels, including immune-related resistance patterns and genomic and gene mapping, to explore the mechanisms underlying immune resistance in EGFR-mutated (mEGFR) NSCLC. These results suggest that the immunologically inert phenotype (low PD-L1 expression, low TMB, and low CD8+ T cells) of mEGFR NSCLC may be attributed to the upregulation of the NT5E (encoding the exonucleosidase CD73) and ADO A1 receptor genes in the ADO pathway. More notably, NT5E was shown to be highly expressed in tumor samples relative to normal lung epithelial cells (59), as well as in TKI-resistant tumor samples compared to untreated tumor samples (12). Griesing et al. (61) and Han et al. (62) previously confirmed the higher expression of CD73 in mEGFR NSCLC using a similar approach. Further, Jin et al. found that CD73 is commonly upregulated in NSCLC. Interestingly, several studies have found that CD73 expression in NSCLC positively correlates with PD-L1 expression (63). In addition, CD73 expression was found to be reduced in EGFR TKI-sensitive cell lines after EGFR TKI treatment (61, 62, 64). However, in EGFR TKI-resistant cell lines, CD73 expression increased and was no longer affected by EGFR TKI treatment (61). This phenomenon has also been observed in clinical specimens (53). In addition, *in vitro* experiments by Le et al. revealed that CD73 is highly expressed on the surface of lung adenocarcinoma cell lines carrying EGFR mutations (12). In a co-culture system of lung adenocarcinoma cells and peripheral blood mononuclear cells, the combination of anti-CD73 and anti-PD-1 antibodies was found to enhance the IFN-γ-mediated antitumor effects of T cells (30). In addition, the antitumor activity of an anti-CD73 antibody combined with an anti-PD-1 antibody has been validated in transgenic mice carrying mEGFR (12). Specifically, another study showed that the combination of the anti-CD73 antibody olecumab and the anti-PD-L1 antibody durvalumab significantly reduced tumor volume in an NSG mouse model carrying mEGFR NSCLC cells and showed that neither olecumab nor durvalumab alone significantly induced antitumor effects. In addition, the combination of oleclumab and durvalumab was found to significantly increase the proportion and number of infiltrating CD8+ T cells in tumors, while also increasing the levels of IFN-γ and TNF-α (64). A2R has also been proposed as a target of interest. Using a homozygous lung cancer mouse model, Chen et al. demonstrated that A2BR-deficient mice exhibited slower tumor growth and a higher frequency of total tumor-infiltrating CD8+ T cells and tumor antigen-specific CD8+ T cells than wild-type mice. In addition, a higher proportion of IFN-γ-secreting CD8+ T cells was identified in the tumors of A2BR-deficient mice (35).

## 4 Introduction of ADO-related drugs approved for oncology-related clinical trials

There are currently two main classes of ADO-related drugs specifically developed for the treatment of tumors (65) (**Table 1**). The first attenuates the effect of ADO on the immune microenvironment by decreasing its concentration in the TME. The mechanisms of action include the inhibition of ADO synthesis and promotion of ADO metabolism. These are predominantly enzyme inhibitors of the ADO production pathway, including CD39, CD73, and CD38. The second category ameliorates immunosuppression by inhibiting ADO function in the TME. These primarily include ADO receptor inhibitors, such as somatic A2AR and A2BR inhibitors. The ADO-related drugs that have entered the clinical study phase are shown in **Table 1**, in which * indicates drugs that have been studied for lung cancer. As shown in **Table 1**, CD39 inhibitors have not been studied independent of lung cancer. In particular, oleculumabs have recently made breakthroughs in the field of lung cancer.

**Table 1:** ADO-related drugs approved for oncology-related clinical trials.

Targets	pharmaceutical classification	pharmaceutical generic names	Approved cancers	NCT NO.	
**CD39**	**monoclonal antibody**	TTX-030	Solid Tumor	NCT03884556	
			Lymphoma	NCT04306900	
		JS019	Solid Tumor	NCT05374226	
			Lymphoma	NCT05508373	
		SRF617	Solid Tumor	NCT05177770	
			Prostate Cancer	NCT04336098	
		PUR001	Solid Tumor	NCT05234853	
		ES002023	Solid Tumor	NCT05075564	
**CD73**	**monoclonal antibody**	Sym024	Solid Tumor	NCT04672434	
		*TJ004309/Uliledlimab	Solid Tumor	NCT05001347	NCT03835949
		HLX23	Solid Tumor	NCT04797468	
		AK119	Solid Tumor	NCT04572152	NCT05559541
		PT199	Solid Tumor	NCT05431270	
		BMS-986179	Solid Tumor	NCT02754141	
		*MEDI9447/oleclumab	Pancreatic Cancer	NCT04940286	NCT04089553
			Breast Cancer	NCT03616886	
			sarcoma	NCT03875573	
			Solid Tumors	NCT04668300	
			Ovarian Cancer	NCT02503774	
			Prostate Cancer	NCT03267589	
			NSCLC	NCT03381274	
		IBI325	Solid Tumor	NCT05119998	NCT05246995
		JAB-BX102	Solid Tumor	NCT05174585	
		NZV930/SRF373	Advanced Malignancies	NCT03549000	
		CPI-006	Advanced Cancers	NCT03454451	
		IPH5301	Solid Tumor	NCT05143970	
	**Small molecule inhibitor**	LY3475070	Advanced Cancer	NCT04148937	
		AB680	Pancreatic Cancer	NCT04104672	
**CD38**	**monoclonal antibody**	*isatuximab/SAR650984	Hematological Malignancy	NCT01084252	NCT04045795
			Neoplasm	NCT03637764	NCT03733717
			Myeloma	NCT02812706	NCT03319667
			Lymphoma	NCT04763616	NCT03275285
			Leukaemia	NCT01749969	NCT03194867
			Prostate Cancer	NCT03769181	NCT02990338
			NSCLC	NCT02283775	NCT02514668
				NCT02332850	NCT04270409
				NCT02999633	NCT04083898
				NCT03860844	NCT05665140
				NCT03367819	NCT04912427
		CID-103	Myeloma	NCT04758767	
		MOR202/MOR03087	Myeloma	NCT01421186	
		TAK079	Myeloma	NCT03439280	NCT03984097
		At211-OKT10-B10	Myeloma	NCT04579523	
		*daratumumab	Prostate Cancer	NCT03177460	NCT03901963
			Bladder Cancer	NCT03473730	NCT05243342
			Kidney Cancer	NCT00574288	NCT05020236
			Myeloma	NCT02419118	NCT03622775
			Leukemia	NCT04407442	NCT02944565
			Glioblastoma	NCT03004287	NCT03695744
			NSCLC	NCT01998971	NCT02977494
			Pancreatic Cancer	NCT03236428	NCT03477539
			Breast Cancer	NCT04280328	NCT03942224
				NCT05392946	NCT03537599
				NCT04933539	NCT03346135
				NCT04246047	NCT03012880
				NCT04892264	NCT03067571
				NCT04756401	NCT04230304
				NCT04352205	NCT03447808
				NCT03734198	NCT04915248
				NCT03937635	NCT03023423
				NCT04139304	NCT03367819
				NCT04922723	NCT03098550
		STI-6129	Solid Tumor	NCT05584709	NCT05308225
			Myeloma	NCT05565807	NCT05519527
			Leukemia		
**A2AR**	**Small molecule inhibitor**	*AZD4635	Prostate Cancer	NCT04089553	
			NSCLC		
		*PBF-509	NSCLC	NCT02403193	
		CS3005	Advanced Solid Tumor	NCT04233060	
		*NIR178	solid tumors	NCT03207867	
			diffuse large B-cell lymphoma	NCT04895748	
			Renal Cell Cancer	NCT03549000	
			Advanced Malignancies		
		*CPI-444/Ciforadenant	Renal Cell Cancer	NCT02655822	
			Prostate Cancer	NCT04280328	
			Multiple Myeloma	NCT05501054	
			NSCLC	NCT03337698	
			Advanced Cancers	NCT03454451	
		*TT-10	Renal Cell Cancer	NCT04969315	
			Prostate Cancer		
			NSCLC		
		Dexdor	Brain Tumor	NCT04266665	
**A2AR, A2BR**	**Small molecule inhibitor**	*AB928/Etrumadenant	Prostate Cancer	NCT04660812	
			Colorectal Cancer	NCT03720678	
			GastroEsophageal Cancer	NCT05177770	
			NSCLC	NCT04262856	
			Malignancies	NCT03629756	
			Triple-Negative Breast Cancer	NCT03719326	
			Ovarian Cancer	NCT04892875	
			Head and Neck Cancers	NCT03846310	
				NCT04660812	
				NCT04381832	

## 5 Safety and efficacy of ADO-related drugs in lung cancer clinical trials

The Hudson study (NCT03334617) was the first to explore the efficacy of the CD73 monoclonal antibody oleclumab in combination with durvalumab in patients with metastatic NSCLC following failure of PD-1/PD-L1 maintenance therapy (66). The results of this study showed a median progression-free survival (mPFS) of 2.63 months and an overall survival (OS) of 12.08 months in patients with acquired resistance to immunotherapy. Although this data are relatively less favorable compared to those of albumin paclitaxel and docetaxel second-line chemotherapy (mPFS: 4.2 and 3.4 months and mOS: 16.2 and 13.6 months, respectively) (67), this study nevertheless indicates a new direction for patients who are intolerant to second-line chemotherapy. Interim data from the COAST study (68), published in April 2022 are also encouraging. For the first time, this study showed that consolidation therapy with a PD-L1 inhibitor (durvalumab) in combination with a CD73 inhibitor (oleclumab) further improved the clinical outcomes in patients with unresectable stage III NSCLC after radiotherapy. Compared to patients maintained on durvalumab alone, the durvalumab combined with oleclumab group showed a significantly increased objective remission rate (ORR) (17.9% vs. 30.0%), disease control rate (DCR) (16-week DCR rate of 58.2% vs. 81.7%, respectively), and prolonged mPFS (6.3 months vs. not achieved). However, OS data have not yet been published. The incidence of emergency adverse events (EAEs) was similar between the combination and single-agent arms. In the combination group, grade 3 EAEs included coughing (1.7%) and dyspnea (1.7%) (68). The COAST study demonstrated, for the first time, that the clinical efficacy of immunotherapy could be improved by immunomodulation. The increased ORR and prolonged mPFS in the combination arm further provide data to support the further development of the world’s first CD73-related phase III clinical study, PACIFIC-9 (NCT05221840). The phase 2 NeoCOAST study further explored the efficacy and safety of durvalumab in combination with olecumab in the neoadjuvant treatment of NSCLC (69). These findings are highly promising (70, 71). The combination therapy group had significantly higher major pathological remission rates (MPR) (11% vs. 19%) and pathological complete remission rates (PCR) (3.7% vs. 9.5%) than the durvalumab monotherapy group. Regarding safety, the incidence of grade 3 or higher treatment-related adverse events (TRAEs) was 0% and 4.8%, respectively. No AE-related deaths occurred in any of the patients. Based on the results of this study, NeoCOAST-2 (NCT05061550) was applied to assess the safety and efficacy of neoadjuvant durvalumab treatment in combination with chemotherapy, olecumab, and adjuvant therapy in patients with resectable early stage NSCLC. Recruitment is currently underway in Japan. Uliledumab is another CD73 inhibitor with efficacy in NSCLC. A previous trial (NCT04322006) was conducted to evaluate the safety, tolerability, and efficacy of uliledlimumab alone or in combination with the PD-1 inhibitor toripalimab in advanced solid tumors. Of the 19 patients with advanced NSCLC who were not candidates for standard therapy, five achieved partial remission and nine had stable disease, with an ORR of 26% and a DCR of 73.7% (72, 73).

CD38 inhibitors are primarily used for the treatment of myeloma and have shown promising results. CD38 inhibitors do not appear to have a definitive efficacy in lung cancer. Two prior trials (NCT03023423 and NCT03367819) have thus far evaluated the efficacy of the CD38 inhibitors isatuximab and daratumumab, respectively, in combination with PD-1/PD-L1 inhibitors in NSCLC but did not achieve satisfactory results. The NCT03023423 trial investigated the safety and efficacy of atezolizumab alone or in combination with daratumumab in patients with advanced or metastatic NSCLC who did not received immunotherapy (74). These studies found no significant improvement in ORR, clinical benefit rate (CBR), mPFS, or mOS in the combination group compared with the single-agent group (ORR 13% vs 4.3%, respectively; CBR 43.5% vs 52.2%, respectively; mPFS 1.5 months vs 1.7 months, respectively; and mOS not achieved vs 7.1 months, respectively) (74). In terms of safety, 38.6% and 56.8% of patients in both groups experienced grade 3 or higher adverse events. In terms of biomarkers, CD38 expression was generally low in both groups, with mean h-scores of only 26.1 and 28.3, respectively (74). The high level of CD38 expression by immune cells in patients with myeloma may be a possible reason for the difference in the efficacy of CD38 inhibitors in NSCLC. NCT03367819 was discontinued after the interim analysis because of a limited treatment response. The results showed that, in terms of efficacy, no patients with NSCLC treated with a combination of isatuximab and cemiplegia achieved complete remission (CR) or partial remission (PR), 65% maintained stable disease (SD), and the mPFS was 4.01 months (75). Interestingly, combination therapy resulted in a decrease in CD38+ immune cells in the TME and an increase in peripherally activated and cytolytic T cells; however, no significant antitumor activity was observed (75). Overall, 70% of the patients developed TRAEs, 20% of which were grade 3 or higher, indicating that the safety of combination therapy is manageable (75).

The Morpheus study compared objective remission rates and safety of second-line therapy with atezolizumab plus CPI-444 and docetaxel in NSCLC patients who showed disease progression during or after treatment with platinum-based regimens and PD-L1/PD-1 checkpoint inhibitors (76). The ORR was 6.7% and 21.4%, respectively, and the mPFS was 2.3 months and 3.2 months, respectively. mOS has not been reported previously (76). In terms of safety, patients receiving atezolizumab and CPI-444 did not experience grade 5 adverse events or adverse events leading to drug (76). This study demonstrated that atezolizumab plus CPI-444 has a controlled safety profile and preliminary antitumor activity.

PBF-509 is another A2AR inhibitor assessed in clinical studies of NSCLC. One phase I/II study investigated the safety, tolerability, and feasibility of the oral immunosuppressant PBF-509 alone or in combination with the PD-1 inhibitor PDR001 for the treatment of NSCLC (77, 78). The DCRs were 42.9% and 66.7% in the single-agent and combination groups, respectively, with ORRs of 9.5% and 8.3%, mOS of 9.7 and 5.4 months, and mPFS of 3.9 and 2.8 months, respectively (78). The incidences of grade 3 or higher TRAEs in the single-agent and combination groups were 16% and 36%, respectively. The most common TRAE in both groups was nausea (44.0% vs. 28%) (78). This study suggests that PBF-509 has preliminary antitumor activity in NSCLC; however, the efficacy of PBF-509 in combination with PD-1 inhibitors needs to be further confirmed.

From the data published in the above studies (as shown in **Table 2**), it is clear that the combination of ADO inhibitors and PD-1/PD-L1 inhibitors has significant potential to improve the prognosis of NSCLC, with obleclumab, a CD73 monoclonal antibody, being the most promising. These studies provided strong evidence that ADO inhibitors can improve or reverse PD-L1 resistance in these patients.

**Table 2:** Clinical study of ADO-related drugs for lung cancer.

Target	Drugs	Participants	Design	Phase	>3grade AEs	Clinical responses	NCT no	Satus	Time of the latest results
CD73	Oleclumab	Metastatic non-small cell lung cancer Who Progressed on an Anti-PD-1/PD-L1 Containing Therapy	durvalumab combined with oleclumab	II	–	acquired drug resistance group: ORR: 4.2% PFS: 2.63 months OS: 12.08 months	**NCT03334617** (HUDSON)	Recruiting	2020
	oleclumab;	Advanced mEGFR NSCLC who progressed after EGFR-TKI treatment	Arm A oleclumab and osimertinib Arm B oleclumab and AZD4635	Ib/II	19.00%	Arm A: ORR: 19% mPFS: 11.0 months mOS: not reached	**NCT03381274**	Active, not recruiting	2021
	oleclumab	Advanced mEGFR NSCLC	Arm A: Oleclumab Arm B: Oleclumab + durvalumab	I	15.10%	OR: 4 SD: 9	**NCT02503774**	Active, not recruiting	2021
	Oleclumab	Consolidation therapy following cCRT of unresectable, Stage III NSCLC	Control Arm: durvalumab Arm A: durvalumab + oleclumab Arm B: Durvalumab + monalizumab	II	Cough (1.7%); dyspnea (1	Control Arm: ORR: 17.9% mPFS: 6.3 months 12-month PFS rates: 33.9% Arm A: ORR: 30.0% mPFS: not reached 12-month PFS rates: 62.6%	**NCT03822351** (COAST)	Active, not recruiting	2022
	Oleclumab	Consolidation therapy following cCRT of unresectable, Stage III NSCLC	Arm A: Durvalumab + Oleclumab Arm B: Durvalumab + Monalizumab Arm C: Durvalumab + Placebo	III	–	–	**NCT05221840** (PACIFIC-9)	Recruiting	–
	Oleclumab	Neoadjuvant therapy of resectable, early-stage (Stage I [>2cm] to IIIA) NSCLC	Control Arm: Durvalumab Arm A: Durvalumab + Oleclumab Arm B: Durvalumab +Monalizumab Arm C: Durvalumab + Danvatirsen	II	4.80%	Control Arm: MPR: 11% pCR: 3.7% ORR: 7.4% Arm A: MPR: 19% pCR: 9.5% ORR: 4.8%	**NCT03794544** (NeoCOAST)	Completed	2022
	Oleclumab	previously untreated, locally recurrent inoperable or metastatic triple-negative breast cancer	Arm A: Paclitaxel + carboplatin + durvalumab + oleclumab Arm B: Paclitaxel + carboplatin + durvalumab	I/II	–	Arm A: CBR: 42.9% mPFS: 6 months Arm B: CBR: 43.3% mPFS: 7.7 months	NCT03616886	Active, not recruiting	2022
	Oleclumab	Advanced Solid Tumors	Arm A: Oleclumab Arm B: Oleclumab + Durvalumab	I	Arm A: 7% Arm B: 21%	Arm A: mPFS: 1.8 months OS: 6.1 months Arm B: mPFS: 1.8 months OS: 5.6 months	NCT02503774	Completed	2023
	Uliledlimab	Advanced Solid Tumor	Arm A: Uliledlimab Arm B: Uliledlimab + Toripalimab	I/II	–	ORR: 26% DCR: 73.7%	NCT04322006	Recruiting	2022
	Uliledlimab	Advanced Solid Tumor	Experimental Arm: Uliledlimab + Atezolizumab	I	–	ORR: 23% DCR: 46%	NCT03835949	Active, not recruiting	2021
	Daratumumab	advanced or metastatic NSCLC who had previously received treatment other than immunotherapy	Arm A: Atezolizumab Arm B: Atezolizumab and Daratumumab	I/II	56.80%	Arm B: ORR: 4.3% CBR: 52.2% mPFS: 1.7 months mOS: 7.1 months	**NCT03023423**	Completed	2020
	Daratumumab	Multiple Myeloma	Daratumumab + carfilzomib + dexamethasone	I	77%	ORR: 84% mPFS: not reached	NCT01998971	Active, not recruiting	2019
CD38	Isatuximab	Non-small cell lung cancer who progressed on anti-PD-1/PD-L1-containing therapy Non-small Cell Lung Cancer	Phase 2 Cohort B and D: Isatuximab + cemiplima	I/II	20%	CR: 0% PR: 0% SD: 65% mPFS: 4.01 months	**NCT03367819**	Terminated	2022
	Isatuximab	Relapsed/refractory multiple myeloma	Arm A: Isatuximab Arm B: Isatuximab + Dexamethason	I/II	Arm A: 13.8% Arm B: 18.20%	Arm A: ORR: 23.9% mPFS: 4.9 months mOS: 18.9 months Arm B: ORR: 43.6% mPFS: 10.2 months mOS: 17.3 months	NCT01084252	Completed	2021
	Isatuximab	Advanced solid tumors	Isatuximab + Atezolizumab	I/II	9.30%	ORR: 9.5% mPFS: 1.92 months	NCT03637764	Terminated	2022
	Isatuximab	Relapsed multiple myeloma	Arm A: Isatuximab + Carfilzomib + Dexamethasone Arm B: Carfilzomib + Dexamethasone	III	Arm A: 1.08 patient per year Arm B: 0.97 patient per year	Arm A: ORR: 86.6% mPFS: 35.7 months Arm B: ORR: 83.7% mPFS: 19.2 months	NCT03275285	Active, not recruiting	2023
	Isatuximab	Relapsed/refractory multiple myeloma	Isatuximab	Ib	88%	51%	NCT01749969	Completed	2017
	Isatuximab	Relapsed/refractory multiple myeloma	Arm A1 (cHL anti-PD-1/PD-L1 naïve): Isatuximab + Cemiplimab + Radiotherapy Arm A2 (cHL anti-PD-1/PD-L1 progressors): Isatuximab + Cemiplimab + Radiotherapy Arm B (DLBCL): Isatuximab + Cemiplimab Arm C (PTCL): Isatuximab + Cemiplimab	I/II	Arm A1: 5.6%; Arm A2: 8.3%; Arm B: 70.6%; Arm C: 81.8%.	Arm A1: mPFS: 8.38 months Arm A2: mPFS: 8.28 months Arm B: mPFS: 2.37 months Arm C: mPFS: 2.66 months	NCT03769181	Terminated	2022
	Isatuximab	Relapsed/refractory multiple myeloma	Arm A: Pomalidomide + Dexamethasone Arm B: Isatuximab + Pomalidomide + Dexamethason	III	–	Arm A: mPFS: 5.9 months mOS: 17.7 months Arm B: mPFS: 11.1 months mOS: 24.6 months	NCT02990338	Completed	2022
	Isatuximab	Relapsed/refractory multiple myeloma	Isatuximab + pomalidomid + dexamethasone	I	59.60%	ORR: 53.2%; CBR: 72.3%; mPFS: not reached; mOS: not reached	NCT02283775	Completed	2021
	Isatuximab	Refractory multiple myeloma	Isatuximab	I	50.00%	ORR: not reached; DCR: 37.5%; mPFS: 1.6 months; mOS: 10.7 months	NCT02514668	Completed	2021
	MOR03087 (MOR202)	Relapsed/refractory multiple myeloma	Arm A: MOR03087 q2w Arm B: MOR03087 q1w Arm C: MOR03087 + dexamethasone Arm D: MOR03087 + pomalidomide + dexamethasone Arm E: MOR03087 + lenalidomide + dexamethasone	I/II	Arm A: 52% Arm B: 100% Arm C: 83% Arm D: 95% Arm E: 100%	Arm C: mPFS: 8·4 months Arm D: mPFS: 17·5 months Arm E:	NCT01421186	Completed	2020
						mPFS: not reached			
**A2AR**	CPI-444	NSCLC patients who progressed during or after receiving platinum based regimen and PD-L1/PD-1 checkpoint inhibitors	Control Arm: Docetaxel Experimental Arm: Atezolizumab + CPI-444	I/II	–	ORR: 6.7% mPFS: 2.3 months	**NCT03337698** (MORPHEU)	Recruiting	2020
	PBF-509	Previously treated patients with advanced NSCLC	Arm A: PBF-509 Arm B: PBF-509 + PDR001	I/II	36.00%	Arm A: DCR: 42.9% ORR: 9.5% mPFS: 3.9 months mOS: 9.7 months Arm B: DCR: 66.7% ORR: 8.3% mPFS: 2.8 months mOS: 5.4 months	**NCT02403193**	Completed	2022

## 6 Potential biomarkers of the clinical benefit of ICBs in combination with ADO-related drugs for lung cancer

Biomarkers that can predict the clinical efficacy of ICBs in combination with ADO inhibitors are still being explored. Many studies have shown that high CD73 expression in tumor tissues is an indicator of poor prognosis in NSCLC (79–84). The expression levels of CD39 and CD38 were also similar significance (85–88). CD73 expression has been found to be positively correlated with histopathological grade, tumor invasion, and lymph node metastasis (63, 89). Of particular interest is the correlation between CD73 and PD-1/PD-L1 expression (63, 90, 91). The expression of both PD-L1 and CD73 is elevated in drug-resistant NSCLC following treatment with EGFR-TKIs (53). Previous studies have shown that CD73 expression can predict the efficacy of immune checkpoint inhibitors (92). High CD73 expression in NSCLC cells appears to be associated with a better response to ICB treatment (84, 93). In addition, it has also been shown that the ratio of MDSCs to CD39+CD8+ T cells could serve as a potential biomarker to predict the blocking effect of immune checkpoint inhibitors in patients with NSCLC (67, 85). The results of one prior trial (NCT04322006) indicated that co-expression of CD73 and PD-L1 may be a potential biomarker for predicting the efficacy of CD73 inhibitors in combination with ICBs (72). In a study that included 19 patients with advanced NSCLC, the clinical response to uliledolimumab and toripalimab treatment was significantly correlated with CD73 expression in the tumor. Four of 5 PR patients had high CD73 expression (tumor cell or immune cell expression level ≥35%), and 4 of 9 SD patients had high CD73 expression (72). Similar findings have been reported in Neocoast study. In the durvalumab and olecularab-combination group, higher baseline CD73 expression was associated with fewer tumor cells surviving surgery and pathological remission. Upregulation of genes related to B cell activation and antigen presentation was also detected in the combination group of patients with MPR. Combination treatment with durvalumab and oleculum also increased the density of immune effector NKG2A+ cells in the tumor immune microenvironment (70). A phase I study, NCT02503774, also found that the frequencies of CD8+ T cells, PD-L1, and granzyme B were upregulated in five of the six patients treated with the combination of durvalumab and olecumab, in whom biomarkers were detected by biopsy (94). However, the evidence provided by these studies was limited. In the future, randomized controlled studies with larger sample sizes should be performed to explore the benefits of combination therapy. Accurate detection of TME components (e.g., with immune cell fraction assay, immunoreactive molecule assay, tumor cell and immune cell surface molecule expression assay, and TMB), complemented by combination proteomics, genomics, single-cell sequencing, next-generation sequencing, and other technologies, will help to further clarify their potential biomarkers and thus guide the application of combination strategies to more appropriate populations and achieve optimal clinical benefits.

## 7 Conclusion and perspectives

Recently, the specific mechanisms of ADO in the formation of an immunosuppressive microenvironment in lung cancer have been revealed. Current evidence suggests that ADO can cause immune resistance in lung cancer by inducing the formation of an immunosuppressive microenvironment, thereby affecting the antigen presentation process, promoting T-cell rejection and T-cell failure, and interfering with IFN-γ signaling pathway. In contrast, ADO inhibitors play critical roles in the above mentioned segments reversing immune resistance. In the field of lung cancer, ADO inhibitors in combination with ICBs have achieved staged progress, and several phase III clinical trials are currently underway. The importance of ADO inhibitors in inhibiting tumor progression and improving the immunosuppressive microenvironment is becoming clear, especially for the CD73 monoclonal antibody olecumab. However, related research is still nascent and there are still many controversies regarding ADO inhibitors. First, the mechanisms underlying the ADO pathway in lung tumorigenesis and development have not yet been fully elucidated. Secondly, the exact efficacy and adverse effects of ADO inhibitors in NSCLC have not yet been demonstrated in phase III clinical studies. Third, the available clinical data were not sufficient to accurately identify the beneficiary population and the scope of use of ADO inhibitors. Finally, more multicenter, randomized, controlled studies are needed to explore the usage strategies of ADO inhibitors, such as which ADO inhibitors are most effective and how they are combined with ICBs. Future studies should address these questions and explore a broader and brighter future for the development and use of ADO inhibitors to provide valuable opportunities for systemic treatment of NSCLC.

## Author contributions

RW: Data curation, Formal analysis, Visualization, Writing – original draft, Writing – review & editing. ZL: Data curation, Writing – original draft, Writing – review & editing. TW: Formal analysis, Writing – original draft, Writing – review & editing. JZ: Formal analysis, Visualization, Writing – review & editing. JL: Conceptualization, Formal analysis, Funding acquisition, Writing – review & editing. QZ: Conceptualization, Formal analysis, Writing – review & editing.
